# Supplementary material for: The causal role of circulating inflammatory markers in osteoporosis: a bidirectional Mendelian randomized study
Source: Front Immunol. 2024 Jul 18;15:1412298. doi: 10.3389/fimmu.2024.1412298 (PMC11291241; doi:10.3389/fimmu.2024.1412298)
Supplement: Supplementary file 1 [file DataSheet_1.zip › supplementary material/OSTEOPOROSIS-CIM/GCST90274848.tsv.gz_leave_one_out.pdf]

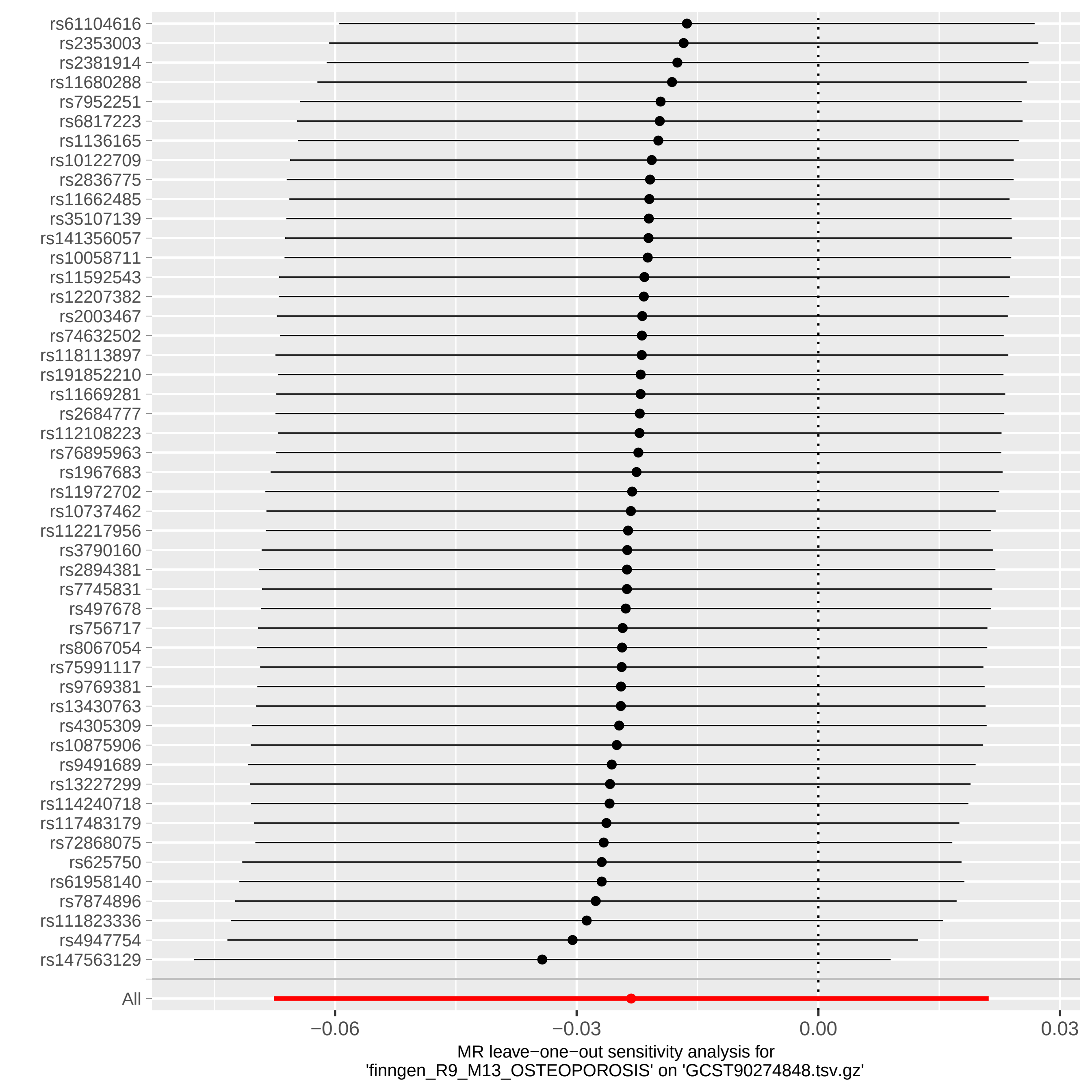
rs61104616
rs2353003
rs2381914
rs11680288
rs7952251
rs6817223
rs1136165
rs10122709
rs2836775
rs11662485
rs35107139
rs141356057
rs10058711
rs11592543
rs12207382
rs2003467
rs74632502
rs118113897
rs191852210
rs11669281
rs2684777
rs112108223
rs76895963
rs1967683
rs11972702
rs10737462
rs112217956
rs3790160
rs2894381
rs7745831
rs497678
rs756717
rs8067054
rs75991117
rs9769381
rs13430763
rs4305309
rs10875906
rs9491689
rs13227299
rs114240718
rs117483179
rs72868075
rs625750
rs61958140
rs7874896
rs111823336
rs4947754
rs147563129
All
−0.06
−0.03
0.00
0.03
MR leave−one−out sensitivity analysis for
'finngen_R9_M13_OSTEOPOROSIS' on 'GCST90274848.tsv.gz'
